# Thank you to our reviewers

**DOI:** 10.1017/cts.2025.16

**Published:** 2025-02-24

We are indebted to the expert referees who have volunteered their time to review submissions for the Journal of Clinical and Translational Science in 2024. The editors, authors, and readers are immensely grateful for their thoughtfulness and expertise that have served our journal well.

Heather Barkholz

Mark B. Marchant

Chad Abresch

Sara L. Ackerman

Stacey Adam

Joan Adamo

Lori Adams

Reuben Adatorwovor

Adebola Adegboyega

Rachel Adler

Prajakta Adsul

Samuel E. Adunyah

Sergio Aguilar-Gaxiola

Gopal Allada

Caitlin Allen

Michele Allen

Cole Allick

Ernest Amankwah

Emily Anderson

Nicki Apaydin

Kat Aribindi

Jennifer Armstrong

Sarah Armstrong

David Asai

Kelly A. Aschbrenner

Simon Ascher

Irfan Asif

Mona AuYoung

Shasha Bai

Andrew Balas

Christian Balbin

Michael Eliot Bales

Lauren Balmert

Shweta Bansal

Claudia Baquet

Rana Barar

Geoanna Bautista

Derek Bays

Michael Becich

Joseph Bednash

Jasmine Bell

Wyatt P. Bensken

Stephen Bergin

Steven L. Bernstein

Nrupen Bhavsar

Jiang Bian

Barbara Bierer

Heather Billings

Suzi Birz

Edward A. Bittner

Allison Black

Arthur Blank

Heather Bleacher

Beatrice A. Boateng

Leanne Boehm

Kostiantyn Botnar

Carolyn Bramante

Allan R. Brasier

Ann Brearley

Miriam A. Bredella

Sarah K. Brewer

James R. Broach

Amanda Marie Brock

Guy Brock

Tabetha A. Brockman

Timothy Brown

Ross Brownson

Donald Bucher

Jessica M. Burke

Thomas Burklow

Elizabeth Burner

John B. Buse

Karen Calhoun

Cliff Callaway

Kenzie A. Cameron

Kendall Campbell

Laura Campbell

Maribel Campos

Ramon Cancino

Marlene Cano

Bernadette Capili

Jennifer Carrera

Savanna Carson

Lisa Carter-Bawa

Lori Carter-Edwards

Louis Carvajal

Shannon Casey

Paige Castro-Reyes

David Center

David Chambers

Redonna Chandler

Alec Chapman

Rita Charon

Jake Chen

Jin Chen

Zhaoyi Chen

Alex C. Cheng

Johanna Chesley

Esther Choo

Terry Church

Lisa Cicutto

Lisa Cleveland

Nichelle L. Cobb

Elizabeth Cohn

Barry Coller

Chiquita Collins

Sarah Cook

Lisa D. Cooper

Erika Crable

Emily Cramer

Jennifer Croker

Grace Cua

Yvette Cuca

John Cullen

Jennifer Cunningham-Erves

Jessica Currier

Carlton Dampier

Yifang Dang

Nisha Datta

Denise Daudelin

Gaurav Dave

Wilfredo De Jesus Monge

Colin A. Depp

Kai Ding

Mary Dolansky

Hayoung Kim Donnelly

Jamie Mihoko Doyle

Ann M. Dozier

Mendy Dunn

Zachary Dyer

Carrie Dykes

Douglas Easterling

Dixie Ecklund

Elizabeth Eckstrom

Milton Mickey Eder

José Eduardo

Sarah Egan

Howard Eisenson

Tim Elgin

Aris Eliades

Peter L. Elkin

Vicki L. Ellingrod

Helena Ellis

Julie T. Elworth

Chrysilla Emanuelle

Felicity T. Enders

Kolaleh Eskandanian

Juan C. Espinoza

Estela Estape

Megan L. Falsetta

Elissa Faro

Steven R. Feldman

Maret Felzein

Andrew Fesnak

Josh Fessel

Cecilia Fochesato

Kristie Foley

Daniel Ford

Mona N. Fouad

Deborah M. Fournier

Leslie Francis

Stephanie Freel

Christopher Frei

Daniela B. Friedman

Al’ona Furmanchuk

Byron Gajewski

Emily Gallagher

Eden Gallegos

Tanja Gangarova

Jennifer Garner

Mia Geisinger

Eman Ghanem

Sue Giancola

Jesse Gibson

Conrad Gleber

Nicola Godfrey

Rachel Gold

Judith D. Goldberg

Mark Goldberg

Ben Goldstein

Brittany Gomez

Mitzi Gonzales

Urszula Górska-Klimowska

Jonathan Green

Harry Greenberg

Kevin Grumbach

Serena Guo

Yawen Guo

Yi Guo

Mikel Gurrea-Rubio

Melissa Haendel

Nathaniel Hafer

Mark Hall

Melissa Hall

Lance Hallberg

Megan E. Hamm

Noah Hammarlund

Daniel F. Hanley

Peggy A. Hannon

Md Rejuan Haque

Brett Harnett

Richart Harper

Paul A. Harris

Lisa Harrison

Jeffrey Hasday

Anuradha Hashemi

Larry W. Hawk, Jr.

Jacqui Hawkins

Sarah Haynes

Helen Hazuda

Shirley L.T. Helm

Edgard Hernandez

Sinsi Hernández-Cancio

Patrice Hicks

William Hogan

Lauren Houston

Carol Howe

Xiaodan Hu

Yan Hu

Yu Huang

Allison Hubel

Tim Huerta

Joe Hunt

Phillip Ianni

David H. Ingbar

Noura Insolera

Tiffany Israel

Rebekah Jacob

Michael A. Jacobs

Laura P. James

Carey Jansen

Esther Jeon

Rebecca N. Jerome

Xingming Jiang

Xia Jing

Brenda Joly

Carolynn Thomas Jones

Hendree Jones

Rebecca Jones

Yvonne C. Jonk

Felix Kabo

A. Kalet

Niranjan Karnik

Heidi Keeler

Patrick William Kelly

Nick Kenyon

Maura M. Kepper

Jared Kerr

Anthony Keyes

Raihan Khan

Sana Khoury-Shakour

Lisa Kilpela

Mimi Y. Kim

Sujin Kim

Judy A. Kimberly

Robert Kimberly

Randall Kimple

Sreelata Kintala

Bernadette Kiraly

Heather Kitzman

Amber Kleckner

Lisa M. Klesges

Jacqueline Knapke

Teresa L. Knott

Angelica Koch

Laura Konig

Kimberly Kopecky

Rhonda G. Kost

Joseph A. Kotarba

Stephanie A. Kraft

Penny Kris-Etherton

marie Krousel-Wood

Dustin Krutsinger

Jack Kues

Polina Kukhareva

Sheila Kusnoor

Paul Kuttner

Hyunjung Kwak

Bethany M. Kwan

Marcus Lambert

Karen Lane

Victor Lara-Díaz

Bernard LaSalle

Jerold Last

Katharine Lawrence

Alana LeBron

MinJae Lee

Rebekka Lee

Diana Lee-Chavarria

Charity Lehn

Jennifer LeLaurin

Vivian Lewis

Chenyu Li

Jenny Libien

Maureen Lichtveld

Junjing Lin

Mike Linke

Janelle LInton

Lipeng Liu

Yiyang Liu

Tammy L. Loucks

Douglas Luke

Hillary A. Lum

Katherine Luzuriaga

XIaomeng Ma

Charisse Madlock-Brown

Zahra Mahamed

Jane Mahoney

Kris M. Markman

Karen G. Martinez

Meghan Martz

Kate Marusina

Colleen A. Mayowski

Stephanie Mazzucca

Joseph McClernon

Wayne T. McCormack

Jennifer B. McCormick

Joseph B. McCormick

Melissa McDaniels

Richard McGee

Christy McKinney

Emma Anne Meagher

Valentina Medici

Jareen Meinzen-Derr

Robin Mermelstein

Eric Robert Messner

Oanh Meyer

Lloyd Michener

Norweeta Milburn

Shirz Mishra

Tessianna A. Misko

Masoud Mohammadnezhad

Sean Mooney

Brioni Moore

Hamidrazi Moradi

Laura E. Moreno

Cynthia Morris

Jason Morrison

Jason Morrow

Ned Mossman

Ahmad Mourad

Jessica Mozersky

Michael Muhammad

Rahma Mungia

Audrey J. Murrell

Kiran Nadndalike

Donald E. Nease

Eric Nehl

Mahdi Neshan

Jason Newland

Michelle Nichols

Keith C. Norris

Wynne Norton

Amy Nowacki

Ucheoma Nwaozuru

Kathleen O’Malley

Ivan Oransky

Mila Ortigoza

Kasim Ortiz

Truls Ostbye

Robert A. Oster

Debra Oto-Kent

Francisco Timbó de Paiva Neto

Marisha E. Palm

Nancy Pandhi

Rechelle Paranal

Hannah Park

Alison Parker

Samuel Thomas Parnell

Sairam Parthasarathy

Kal Pasupathy

Tanha Patel

Cecilia Patino-Sutton

Greg Peck

Clara M. Pelfrey

Jing Peng

Linda Perez-Laras

Susan M. Perkins

Henry B. Perry

Darina V. Petrovsky

Christopher Pittenger

Roy Poblete

Gina-Maria Pomann

Jerlym Porter

Susan Pusek

Borsika A. Rabin

Fornessa Randall

Damayanthi Ranwala

Rhiannon Deierhoi Reed

David Resnik

Staci S. Reynolds

Robert L. Rhyne

Naomi Riches

Rachel Richesson

Marisa Rinkus

Diego Rios-Zertuche

Matthew Rizzo

Donita L. Robinson

Bonny Rockette Wagner

Max Rohde

Cortnee Roman

Rebecca Roper

Gustavo Rosania

Lisa G. Rosas

Jason Rosenfeld

Gary rosenthal

Katie Ross-Driscoll

Reza Rostami

Amihai Rottenstreich

Stephanie Rowan

Doris Rubio

Michael Rubyan

Michael Rudolph

Yin Rui

Neil Ryan

Roy T. Sabo

Malarkodi Jebathilagam Samayamuthu

Elias Samuels

Krista Scheffey

Susanne Schmidt

Sarah Schmiege

Robert Schnoll

Nancy Schwartz

Peter Schwartz

Andiara Schwingel

Harry Selker

Rebecca Selove

Raj C. Shah

Jackilen Shannon

Eugene D. Shapiro

Laura Aubree Shay

Payam Sheikhattari

Gan Shi

Matisyahu Shulman

Mina Silberberg

Tamara Simon

Elise Smith

Jessica Snowden

Denise C. Snyder

Julian Solway

Christine Sorkness

Marisa Spann

Jessica Sperling

Linda Susan Sprague Martinez

Heidi M. Spratt

Lindsay Squeglia

Jack Stapleton

Justin Starren

Anna Steeves-Reece

Mark Stein

Kathleen R. Stevens

Eric strobl

Heidi Sucharew

Yi Sun

Manu S. Sundaresan

Nicole Sur

Zul Surani

Hilary L. Surratt

Jeffrey Szychowski

Teresa Taft

Meagan Talbott

Melanie Taverna

Bradley Taylor

Sandra Taylor

Jessie Tenenbaum

Diana Thomas

Gelise Thomas

Neena Thomas

George Thompson

Victoria Tiasse

Beth B. Tigges

Joan Pennington Totka

Anne Trontell

Joel Tsevat

Kristin Tully

Trevor Tuma

Laurene Tumiel Berhalter

Jason Umans

Heatherlun Uphold

David Ussery

Sangeetha Vadakke-Madathil

Gayle Valensky

Robin Vanderpool

Eleni Okeanis Vaou

Aditi Vasan

Elizabeth Vasile

Evelyn Vázquez

Nadja Vielot

Clare Viglione

Aubrey Villalobos

Alfred Vitale

Boris Volkov

Sarah Vordenbergy

Enya Vroom

Russ Waitman

Nina Wallerstein

Yan Wang

Yanfei Wang

Yanshan Wang

Molly Wasko

Fern Webb

Paul Weber

Lai Wei

Lisa C. Welch

Leah J. Welty

Jack Westfall

Christopher Wheldon

Mark Wieland

Erin Wieruszewski

Christopher Williams

Charlton Wilson

Karen Wilson

Eli Wisdom

Nicole Wolfe

Julian Wolfson

Kevin Wooten

Yonghui Wu

Yaomin Xu

Jade Yang

Matheos Yosef

Reza Yousefi Nooraie

Fei Yu

Sonia Zarate

Fangda Zhang

Lixin Zhang

Qi Zhang

Runzhi Zhang

Xinyi Zhang

Yiye Zhang

Yi Zheng

Shujin Zhong

Monica Zigman Suchsland

Emily B. Zimmerman

Carmen Zorrilla

